# Discrete Virus Factories Form in the Cytoplasm of Cells Coinfected with Two Replication-Competent Tagged Reporter Birnaviruses That Subsequently Coalesce over Time

**DOI:** 10.1128/JVI.02107-19

**Published:** 2020-06-16

**Authors:** Elle A. Campbell, Vishwanatha R. A. P. Reddy, Alice G. Gray, Joanna Wells, Jennifer Simpson, Michael A. Skinner, Philippa C. Hawes, Andrew J. Broadbent

**Affiliations:** a The Pirbright Institute, Woking, United Kingdom; b Section of Virology, Faculty of Medicine, Imperial College London, London, United Kingdom; St. Jude Children’s Research Hospital

**Keywords:** IBDV, birnavirus, coinfection, double-stranded RNA virus, reassortment, viroplasm, virus factory

## Abstract

The *Birnaviridae* family, responsible for major economic losses to poultry and aquaculture, is composed of nonenveloped viruses with a segmented double-stranded RNA (dsRNA) genome that replicate in discrete cytoplasmic virus factories (VFs). Reassortment is common; however, the underlying mechanism remains unknown given that VFs may act as a barrier to genome mixing. In order to provide new information on VF trafficking during dsRNA virus coinfection, we rescued two recombinant infectious bursal disease viruses (IBDVs) of strain PBG98 containing either a split GFP11 or a tetracysteine (TC) tag fused to the VP1 polymerase (PBG98-VP1-GFP11 and PBG98-VP1-TC). DF-1 cells transfected with GFP1-10 prior to PBG98-VP1-GFP11 infection or stained with a biarsenical derivative of the red fluorophore resorufin (ReAsH) following PBG98-VP1-TC infection, had green or red foci in the cytoplasm, respectively, that colocalized with VP3 and dsRNA, consistent with VFs. The average number of VFs decreased from a mean of 60 to 5 per cell between 10 and 24 h postinfection (hpi) (*P* < 0.0001), while the average area increased from 1.24 to 45.01 μm^2^ (*P* < 0.0001), and live cell imaging revealed that the VFs were highly dynamic structures that coalesced in the cytoplasm. Small VFs moved faster than large (average 0.57 μm/s at 16 hpi compared to 0.22 μm/s at 22 hpi), and VF coalescence was dependent on an intact microtubule network and actin cytoskeleton. During coinfection with PBG98-VP1-GFP11 and PBG98-VP1-TC viruses, discrete VFs initially formed from each input virus that subsequently coalesced 10 to 16 hpi, and we speculate that *Birnaviridae* reassortment requires VF coalescence.

**IMPORTANCE** Reassortment is common in viruses with segmented double-stranded RNA (dsRNA) genomes. However, these viruses typically replicate within discrete cytoplasmic virus factories (VFs) that may represent a barrier to genome mixing. We generated the first replication competent tagged reporter birnaviruses, infectious bursal disease viruses (IBDVs) containing a split GFP11 or tetracysteine (TC) tag and used the viruses to track the location and movement of IBDV VFs, in order to better understand the intracellular dynamics of VFs during a coinfection. Discrete VFs initially formed from each virus that subsequently coalesced from 10 h postinfection. We hypothesize that VF coalescence is required for the reassortment of the *Birnaviridae*. This study provides new information that adds to our understanding of dsRNA virus VF trafficking.

## Abstract (precis)

Reassortment is common in viruses with segmented double-stranded RNA (dsRNA) genomes. However, these viruses typically replicate within discrete cytoplasmic virus factories (VFs) that may represent a barrier to genome mixing. We generated the first replication competent tagged reporter birnaviruses, infectious bursal disease viruses (IBDVs) containing a split GFP11 or tetracysteine (TC) tag and used the viruses to track the location and movement of IBDV VFs, in order to better understand the intracellular dynamics of VFs during a coinfection. Discrete VFs initially formed from each virus that subsequently coalesced from 10 h postinfection. We hypothesize that VF coalescence is required for the reassortment of the *Birnaviridae*. This study provides new information that adds to our understanding of dsRNA virus VF trafficking.

## INTRODUCTION

Members of the *Birnaviridae* family are responsible for some of the most economically devastating diseases to the poultry industry and aquaculture: infectious bursal disease virus (IBDV) is endemic worldwide and ranks in the top five diseases of chickens in nearly all countries surveyed (1). As well as causing severe morbidity and mortality, the virus is immunosuppressive, leaving birds that recover with an increased susceptibility to secondary infection and a reduced response to vaccination programs (2, 3). Infectious pancreatic necrosis virus (IPNV) is responsible for high mortalities in farmed salmon and trout and some strains can cause persistent infection, with fish spreading the virus by vertical or horizontal transmission (4). In addition, more recently described birnaviruses, for example, chicken proventriculus necrosis virus (5) and blotched snakehead virus (6), cause production loses that are only just beginning to be understood, and birnaviruses of insects such as Drosophila X virus and Culex Y virus are useful as tools for studying cellular antiviral responses (7). However, despite the importance of these viruses, our understanding of how they replicate in cells is lacking.

The *Birnaviridae* genome is comprised of two segments of double-stranded RNA (dsRNA). Segment A encodes two overlapping reading frames (ORFs), one encoding a nonstructural protein (termed VP5 in IBDV and IPNV) and the other a polyprotein that is cleaved into the capsid protein (VP2), protease (VP4), and a dsRNA binding protein (VP3). Segment B contains one ORF encoding an RNA-dependent RNA polymerase (VP1). Some VP1 copies bind the 5′ and 3′ ends of each genome segment and are packaged into the virion. The dsRNA genome is coated with VP3, which binds VP1 and activates its polymerase activity (8). Collectively, VP1, VP3, and the dsRNA genome form a viral ribonucleoprotein (vRNP) complex (9).

The virus enters host cells by endocytosis or macropinocytosis. As the calcium concentration in the endosome drops, the virus uncoats, releasing a peptide that leads to the formation of pores in the endosomal membrane (10). It is thought that the vRNP complexes exit the endosome through the pores to initiate transcription and translation and form a replication complex, or virus factory (VF) by coopting endosomal membrane components (11, 12). Unlike *Reoviridae*, which are also nonenveloped viruses with a segmented dsRNA genome, the *Birnaviridae* possess only one capsid and lack a transcriptionally active T2 core. It has been demonstrated that genome replication does not require the presence of the capsid (9, 13), and it is thought that the proteins in the vRNP complex shield the dsRNA genome from the cellular sensing machinery (14).

Little is known regarding the number, location, and dynamics of birnavirus VFs during an infection. In contrast, the VFs of mammalian orthoreovirus are known to be highly dynamic, moving in the cytoplasm in a microtubule-dependent manner (15). This is, in part, due to a lack of tagged reporter birnaviruses. Recently, a split green fluorescent protein (GFP) system has been used to generate tagged reporter viruses (16–19). Briefly, the nucleotide sequence encoding the 11th beta sheet of the GFP molecule (GFP11) is incorporated into the viral genome such that a viral protein tagged with the GFP11 peptide is translated. A plasmid encoding the rest of the protein (GFP1-10) is transfected into cells, and when the two come together, they produce a complete, fluorescing GFP. A tetracysteine (TC) tag has also been used to engineer tagged reporter viruses (15, 20–26). Briefly, the TC tag is comprised of 6 amino acids, including two separate pairs of cysteine residues (CCPGCC). When live infected cells are stained with biarsenical compounds (for example, FLAsH-EDT_2_ or ReAsH-EDT_2_), the compounds covalently bind to the cysteine residues in the TC tag. This interaction leads to FLAsH-EDT_2_ fluorescing green and ReAsH-EDT_2_ fluorescing red (27). Here, we describe the generation of the first ever replication-competent tagged reporter birnaviruses, IBDVs tagged with either GFP11 or TC, and we use the viruses to describe the location and movement of *Birnaviridae* VFs in the cytoplasm.

Since the *Birnaviridae* genome is divided into two segments, reassortment is a problem in the field. Reassortment of IBDV has been observed to occur between field strains and vaccine strains (28), between different serotypes (29), and is thought to be responsible for the emergence of very virulent strains (30), and reassortment has also been observed during IPNV infections (4, 31), complicating the epidemiology of the diseases. However, despite the importance of reassortment, the molecular mechanisms involved remain unknown. In order for reassortment to occur, the same cell must become coinfected and the viral gene segments must reach the same intracellular compartment. Because the *Birnaviridae* replicate within discrete VFs, which may be a barrier for genome mixing, it remains unknown how genome segments reach the same intracellular compartment. Here, we track the location and movement of *Birnaviridae* VFs during coinfection of DF-1 cells with GFP11- and TC-tagged IBDVs in order to better understand the intracellular compartmentalization of VFs from two different strains of dsRNA virus during a coinfection and therefore the potential for reassortment throughout the replication cycle.

## RESULTS

### Construction of tagged reporter IBDV strains.

**FIG 1:**
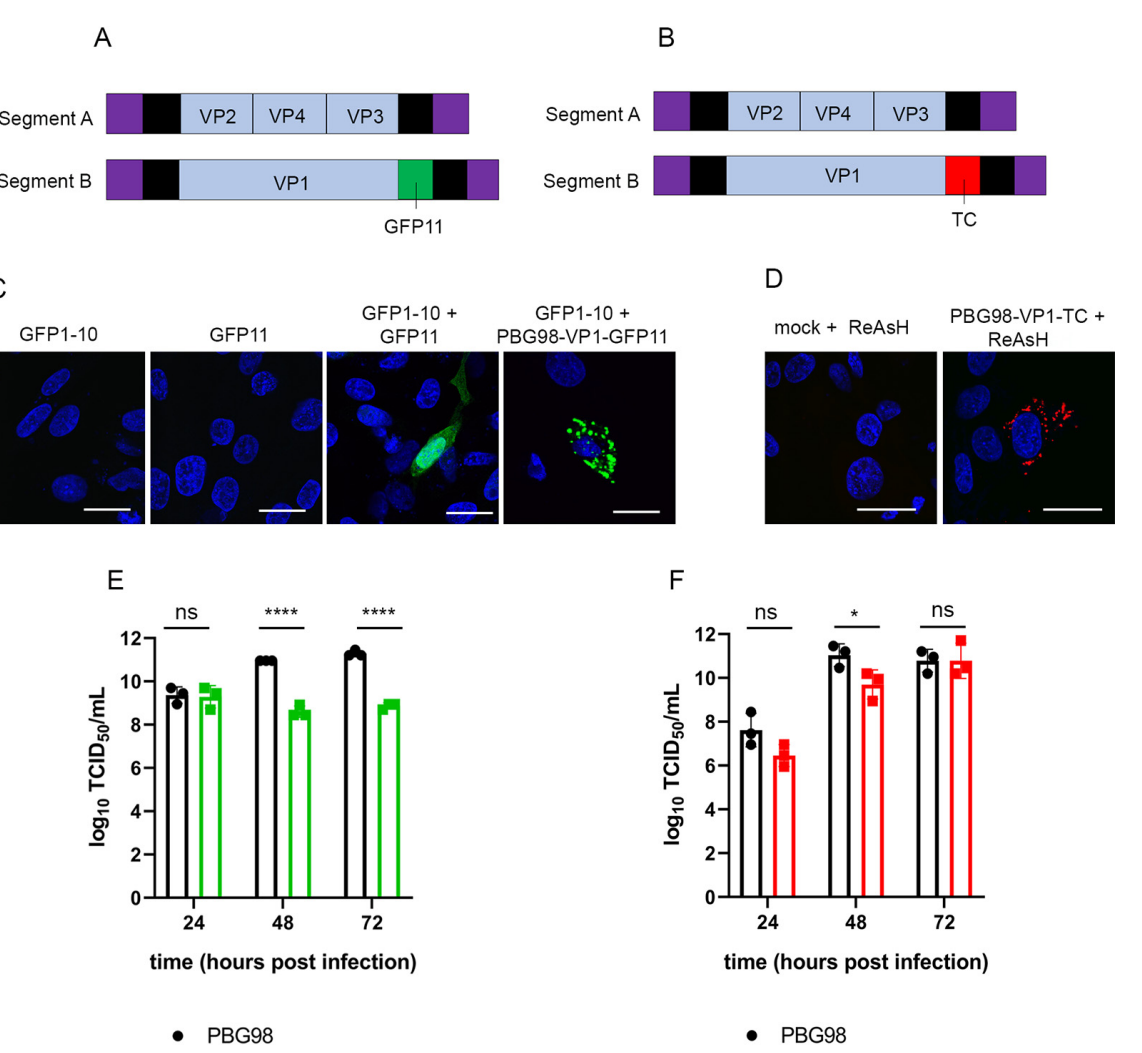
Construction of tagged reporter IBDV strains. The PBG98 sequence was used as a backbone, and the nucleotide sequences encoding either the GFP11 tag (A) or TC tag (B) were added to the 3′ end of the coding region of segment B. (C) To obtain a positive GFP signal, DF-1 cells were transfected with a plasmid expressing GFP1-10 prior to transfection with a plasmid expressing GFP11 or infection with the PBG98-VP1-GFP11 virus. (D) To visualize the TC tag, DF-1 cells infected with the PBG98-VP1-TC virus were stained with ReAsH. The replication of both tagged viruses in DF-1 cells was compared to the recombinant wild-type (wt) PBG98 virus. The titer to which the GFP11-tagged virus (E) or TC-tagged virus (F) replicated was compared to the wt PBG98 virus at the indicated time points postinfection in triplicate. Virus titers were expressed as the log_10_ TCID_50_/ml, individual replicates were plotted as a scatterplot, and the means were plotted as a bar graph with error bars representing the standard deviations (SD) of the mean. The data are representative of three independent experiments. ns, not significant; *, *P* < 0.05; ****, *P* < 0.0001.

A reverse genetics system developed in our laboratory was used to rescue a cell-culture-adapted IBDV strain, PBG98. The PBG98 sequence was used as a backbone, and the nucleotide sequences encoding either the GFP11 or TC tags were added to the 3′ end of the coding region of segment B, which encodes the VP1 polymerase (Fig. 1A and B). Cells were transfected with the GFP1-10 molecule prior to PBG98-VP1-GFP11 infection, which allowed a full-length GFP protein to assemble in the cytoplasm, tagged to the C terminus of VP1. Multiple green foci were observed in the cytoplasm of infected cells, in contrast to cells transfected with GFP1-10 or GFP11 alone, which showed no positive signal, or cells transfected with GFP1-10 and subsequently transfected with a plasmid encoding GFP11, where a positive signal was observed throughout the cell (Fig. 1C). To visualize the TC tag, cells infected with the PBG98-VP1-TC virus were stained with a biarsenical derivative of the red fluorophore resorufin (ReAsH). Multiple red foci were observed in the cytoplasm of infected cells, in contrast to mock-infected cells also treated with ReAsH (Fig. 1D). The replication of both tagged viruses in DF-1 cells was compared to the recombinant wild-type (wt) PBG98 virus. In one experiment, the PBG98 virus replicated to a peak titer of 11.3 log_10_ 50% tissue culture infective doses (TCID_50_)/ml at 72 h postinfection (hpi), whereas the PBG98-VP1-GFP11 virus only reached a titer of 8.9 log_10_ TCID_50_/ml (*P* < 0.0001) (Fig. 1E). In another experiment, the PBG98 virus replicated to a titer of 10.8 log_10_ TCID_50_/ml at 72 hpi that was matched by the PBG98-VP1-TC virus, although replication was lower at 48 hpi (*P* < 0.05) (Fig. 1F). Taken together, both tagged viruses were attenuated, but the TC-tagged virus was less attenuated than the GFP11-tagged virus. The stability of the tags was determined by serially passaging the tagged viruses ten times in DF-1 cells and then imaging cells infected with the supernatants from each passage that either expressed GFP1-10 or were stained with ReAsH. The PBG98-VP1-GFP11 virus was stable up to seven passages, whereas the TC tag continued to be expressed even at passage 10, demonstrating that the PBG98-VP1-TC strain was more stable than the PBG98-VP1-GFP11 virus (data not shown).

### The PBG98-VP1-GFP signal is a marker for IBDV VFs.

**FIG 2:**
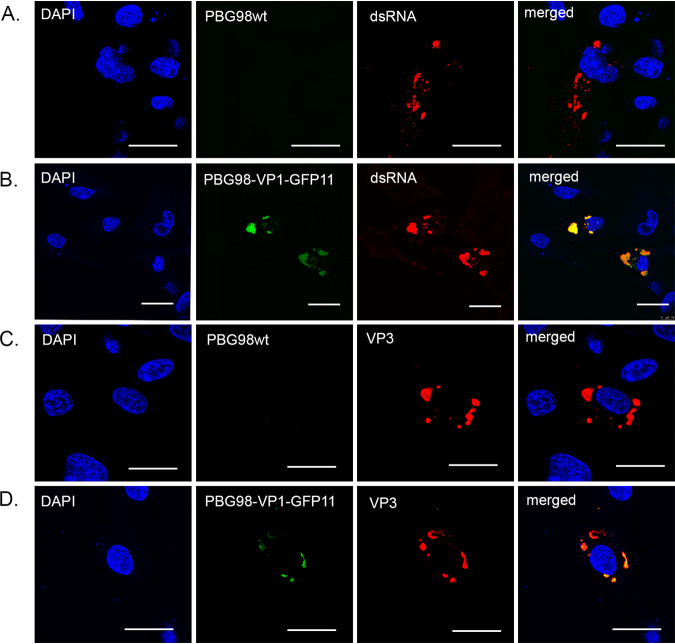
The PBG98-VP1-GFP signal is a marker for IBDV VFs. DF-1 cells were either infected with the recombinant wild-type (wt) PBG98 virus (A and C) or transfected with a plasmid expressing GFP1-10 and infected 24 h posttransfection with the PBG98-VP1-GFP11 virus (B and D) at an MOI of 1. Cells were fixed at 20 hpi and stained with DAPI and either anti-dsRNA (A and B) or anti-VP3 (C and D) mouse monoclonal antibodies, followed by a goat anti-mouse secondary antibody conjugated to Alexa Flour 568, and then imaged. Scale bars, 20 μm.

**FIG 3:**
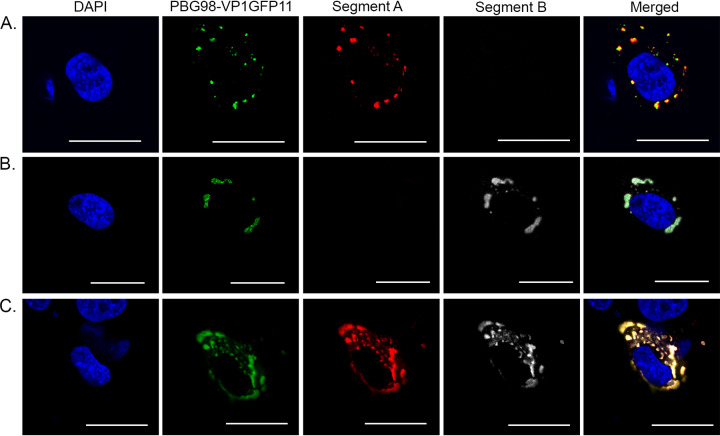
RNA from both segments A and B are located within the VFs. DF-1 cells were transfected with a plasmid expressing GFP1-10 and infected 24 h posttransfection with the PBG98-VP1-GFP11 virus at an MOI of 1. Cells were fixed at 24 hpi, and 48 Stellaris FISH probes conjugated to Quasar 570 dye were hybridized to RNA from segment A (A), 48 Stellaris FISH probes conjugated to Quasar 670 dye were hybridized to RNA from segment B (B), or a cocktail of the 96 probes were cohybridized (C). Scale bars, 20 μm.

DF-1 cells were either infected with the recombinant wt PBG98 virus or transfected with GFP1-10 and infected with the PBG98-VP1-GFP11 virus. Infected cells were fixed at 20 hpi and stained with either anti-dsRNA or anti-VP3 mouse monoclonal antibodies, followed by a goat anti-mouse secondary antibody conjugated to Alexa Flour 568 (goat anti-mouse 568) (Fig. 2). The GFP11 signal was highly colocalized with both dsRNA and VP3 (Fig. 2B and D); for example, 82.6 ± 0.1% of the signal derived from PBG98-VP1-GFP11 colocalized with the VP3 signal. These data demonstrate that the PBG98-VP1-GFP11 signal colocalized with other vRNP components, consistent with the fluorescent signal from the tagged viruses being a marker for VFs. The approximate size and distribution of the VP3 and dsRNA signal was the same in cells infected with the wt virus or the tagged virus, suggesting that the addition of the tag did not affect VF morphology or location. DF-1 cells transfected with GFP1-10 and infected with the PBG98-VP1-GFP11 virus were also subject to fluorescent *in situ* hybridization (FISH) at 24 hpi, with probes designed to bind mRNA encoded by PBG98 segments A or B (Fig. 3). Probes were specific, with no cross-reactivity to the other segment, and the signal from both probes colocalized with the VFs, suggesting that viral mRNA is restricted to the VFs and not elsewhere in the cytoplasm.

### VFs coalesce in the cytoplasm of infected cells throughout infection.

**FIG 4:**
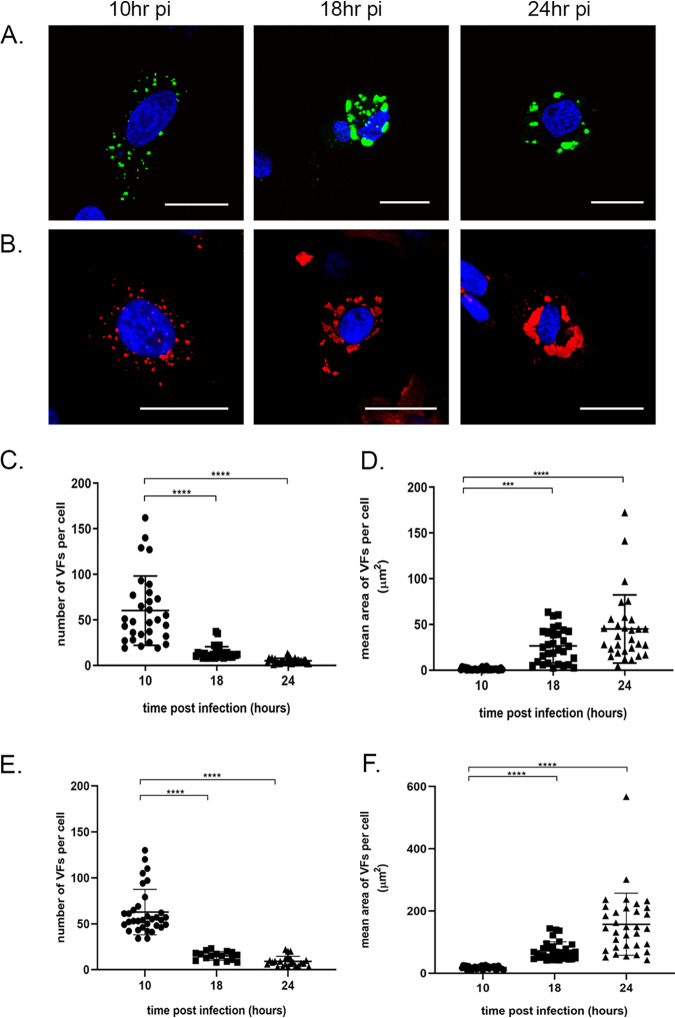
VFs decrease in number and increase in size over time. DF-1 cells were either transfected with GFP1-10 and infected with the PBG98-VP1-GFP11 virus (A) or infected with the wt PBG98 virus (B) an MOI of 1 and fixed at 10, 18, and 24 hpi, stained with DAPI, and imaged. The wt PBG98-infected cells were also stained with mouse anti-VP3 and goat anti-mouse-568 for visualization of VFs. Scale bars, 20 μm. The number of VFs per cell was determined for 30 infected cells at each time point and plotted for cells infected with the PBG98-VP1-GFP11 virus (C) and the wt PBG98 virus (E). The average area of the VFs in an infected cell was determined using the surface tool in Imaris 9 software (bitplane) and plotted for 30 infected cells at each time point for cells infected with the PBG98-VP1-GFP11 virus (D) and the wtPBG98 virus (F). The line represents the mean, and the error bars represent the SD of the mean. The numbers and sizes of foci were compared using Kruskal-Wallis one-way ANOVA. ***, *P* < 0.001; ****, *P* < 0.0001.

DF-1 cells were either infected with the wt PBG98 virus or transfected with GFP1-10 and infected with the PBG98-VP1-GFP11 virus at an MOI of 1 and fixed at 10, 18, and 24 hpi. The wt PBG98- infected cells were stained with mouse anti-VP3 and goat anti-mouse-568 for visualization of VFs. At early time points, distinct, small foci were abundant throughout the cytoplasm of infected cells; however, at later time points the foci were larger in size and fewer in number. This was true for cells infected with the tagged virus (Fig. 4A) and the wt virus (Fig. 4B). To quantify this observation, 30 infected cells were imaged per time point. The mean number of foci significantly decreased from 60 per cell at 10 hpi to 5 per cell at 24 hpi in cells infected with the PBG98-VP1-GFP11 virus (*P* < 0.0001) (Fig. 4C) and from 62 to 9 in cells infected with the wt PBG98 virus (*P* < 0.0001) (Fig. 4E). In contrast, the average area of each VF significantly increased from a mean of 1.2 μm^2^ at 10 hpi to 45.0 μm^2^ at 24 hpi in cells infected with the PBG98-VP1-GFP11 virus (*P* < 0.0001) (Fig. 4D) and from 17.9 to 158 μm^2^ in cells infected with the wt PBG98 virus (*P* < 0.0001) (Fig. 4F). Taken together, these data suggest that *Birnaviridae* VFs coalesced in the cytoplasm throughout infection in cells infected with both tagged and wt viruses, although the VFs were smaller in cells infected with the tagged virus. As multiple VFs were observed in infected cells, we sought to determine whether this was a feature of multiplicity of infection (MOI). To this end, we infected DF-1 cells with the PBG98-VP1-GFP11 virus at an MOI of 0.1, 0.01, or 0.001 and fixed and imaged cells 18 hpi. Multiple VFs were observed in infected cells, irrespective of the MOI, indicating that this phenomenon was observed even in cells infected with a small amount of infectious virus (data not shown).

**FIG 5:**
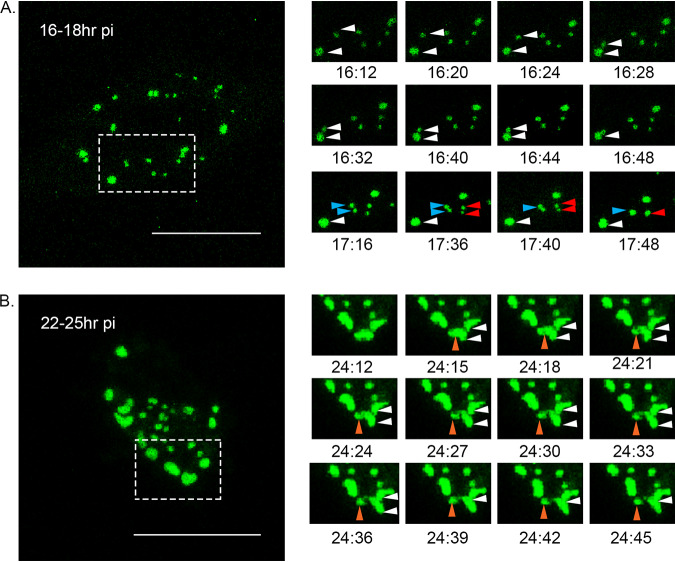
VFs coalesce in the cytoplasm of infected cells throughout infection. DF-1 cells were transfected with a plasmid expressing GFP1-10 and infected 24 h posttransfection with the PBG98-VP1-GFP11 virus at an MOI of 1. (A) One live infected cell was imaged every 4 min from 16 to 18 hpi. Images of the boxed region are shown at the indicated time points postinfection, where three VF coalescence events were witnessed (white, blue, and red arrows). (B) Another live infected cell was imaged from 22 to 25 hpi. Images of the boxed region are shown at the indicated time points postinfection where one VF fusion event was witnessed (white arrows) and one VF fission event was witnessed (orange arrows).

Cells transfected with GFP1-10 and infected with the PBG98-VP1-GFP11 virus were subjected to live cell imaging to investigate the nature of VF movement. Cells were imaged every 4 min over a 2-h period from 16 to 18 hpi and a movie was made (Fig. 5A and see Movie S1 in the supplemental material). Five fusion events were observed between foci in a single cell over this time frame (examples are shown in Fig. 5A). No fission events were noted. Five fusion events were also apparent in cells imaged 22 to 25 hpi (Fig. 5B and Movie S2), although four of these were transient. One smaller focus was also observed splitting away from a larger focus (Fig. 5B and Movie S2). In addition, there was a difference in the speed of focus movement between 16 and 22 hpi. At 16 hpi, foci moved with an average speed of 0.57 μm/s, while at 22 hpi, the foci only moved with an average speed of 0.22 μm/s. Taken together, these data demonstrate that *Birnaviridae* VFs are dynamic structures that coalesce in the cytoplasm.

### VF coalescence is dependent on an intact microtubule network and actin cytoskeleton.

**FIG 6:**
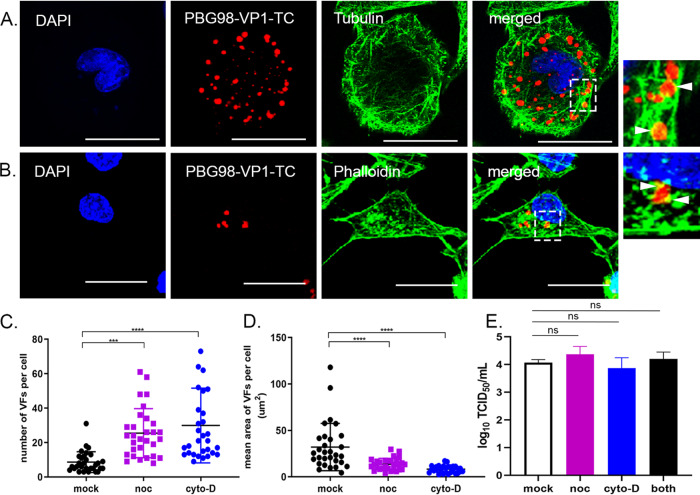
The distribution of VFs in the cytoplasm is dependent on an intact actin cytoskeleton and microtubule network, but alterations in VF distribution do not alter viral replication. DF-1 cells were infected with the PBG98-VP1-TC virus at an MOI of 1. Cells were fixed at 20 hpi and stained with DAPI and either an anti-tubulin mouse monoclonal antibody, followed by a goat anti-mouse secondary antibody conjugated to Alexa Flour 488 to visualize the microtubule network (A) or phalloidin conjugated to Alexa Flour 488 to visualize the actin cytoskeleton (B). Scale bars, 20 μm. Images of the boxed regions are shown enlarged, and the locations where the cytoskeleton colocalized with the VFs are indicated by white arrows. (C) The number of VFs per cell was determined for 30 infected cells and plotted. (D) The average area of the VFs in an infected cell was determined using the surface tool in Imaris 9 software (bitplane) and plotted for 30 infected cells. The line represents the mean, and the error bars represent the SD of the mean. (E) Cell supernatants obtained 24 hpi were titrated, and the TCID_50_ values were determined. Virus titers were compared by one-way ANOVA and a Tukey’s multiple-comparison test following a Shapiro-Wilk normality test to confirm whether the data followed a normal distribution for parametric or nonparametric testing. The mean area and number of VFs were compared using Kruskal-Wallis one-way ANOVA. ns, not significant; ***, *P* < 0.001; ****, *P* < 0.0001.

**FIG 7:**
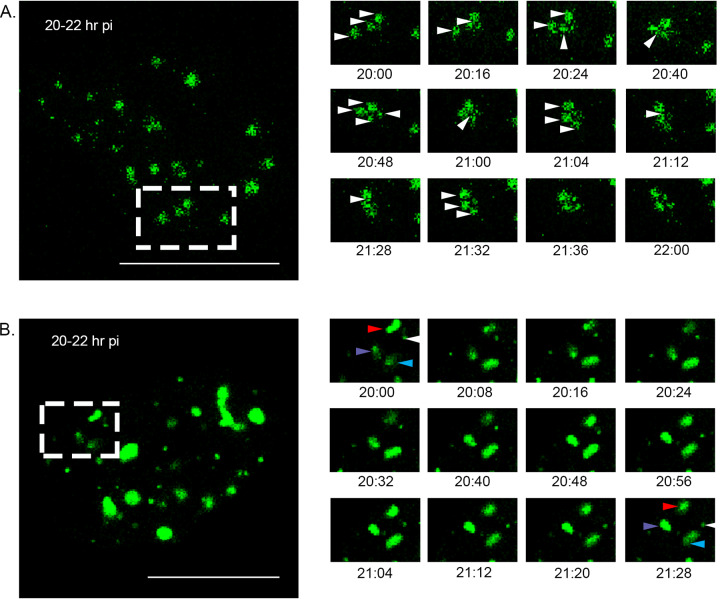
VF coalescence is dependent on an intact microtubule network and actin cytoskeleton. DF-1 cells were transfected with a plasmid expressing GFP1-10 and infected 24 h posttransfection with the PBG98-VP1-GFP11 virus at an MOI of 1. Cells were treated with either nocodazole (A) or cytochalasin-D (B) from 2 hpi. One live infected cell was imaged every 4 min from 20 to 22 hpi. Images of the boxed region are shown at the indicated time points postinfection. (A) In an infected cell treated with nocodazole, VFs were witnessed transiently interacting (white arrows). (B) In an infected cell treated with cytochalasin-D, four VFs were observed (white, blue, red, and purple arrows) that did not coalescence or interact with each other over the 2-h period. Scale bars, 20 μm.

Cells were infected with the PBG98-VP1-TC virus and then stained with ReAsH and either fixed and stained with anti-tubulin and a secondary antibody conjugated to Alexa Flour 488 (Fig. 6A) or phalloidin-Alexa Fluor 488 (Fig. 6B). The edges of the red foci colocalized with both tubulin and phalloidin (Fig. 6A and B, boxed regions and white arrows), suggesting that actin filaments and the microtubule network may be involved in VF movement. To determine whether these structures were involved in *Birnaviridae* VF coalescence, cells were treated with either nocodazole, which triggers microtubule depolymerization, or cytochalasin D, which inhibits actin filament polymerization, 2 h after infection with the PBG98-VP1-TC virus. Actin is known to play a role in IBDV entry (32), and so this time point was selected to distinguish the effect of drug treatment on VF movement from virus entry. At 18 hpi, both nocodazole and cytochalasin D treated cells had significantly more foci per cell (means of 25 and 30 foci per cell, respectively) than mock-treated infected cells (mean of 7 foci per cell; *P* < 0.0001) (Fig. 6C). Nocodazole and cytochalasin D treatments also led to a significantly reduced area of foci at this time point (14.4 and 8.1 μm^2^, respectively) compared to mock-treated infected controls (32.1 μm^2^; *P* < 0.0001) (Fig. 6D). Despite the differences in VF size and number following treatment, neither drug caused significant differences in the titer of the PBG98-VP1-TC virus recovered from the supernatant of infected cultures at 24 hpi (Fig. 6E). Cells were transfected with GFP1-10 and infected with PBG98-VP1-GFP11 at an MOI of 1, treated with either nocodazole or cytochalasin D at 2 hpi, and imaged live from 20 to 22 hpi. In cells treated with nocodazole, foci were seen to go through a series of transient fusion events, at speeds comparable to when no drug treatment was applied (0.62 μm/s). Seven fusion events were observed, all transient in nature, and the foci did not coalesce into larger foci over this time period (Fig. 7A and Movie S3). In contrast, while foci continued to move in cells treated with cytochalasin D, the speed of movement was significantly reduced compared to mock-treated infected cells, with foci moving with a mean speed of only 0.17 μm/s (*P* < 0.0001) (Fig. 7B and Movie S4). Three fusion events were observed over the time frame imaged, and one fission event. Taken together, these data demonstrate that birnavirus VF coalescence is dependent on both an intact microtubule network and actin cytoskeleton and that inhibition of actin polymerization significantly slows birnavirus VF movement.

### Discrete VFs form in the cytoplasm of coinfected cells that coalesce throughout infection.

**FIG 8:**
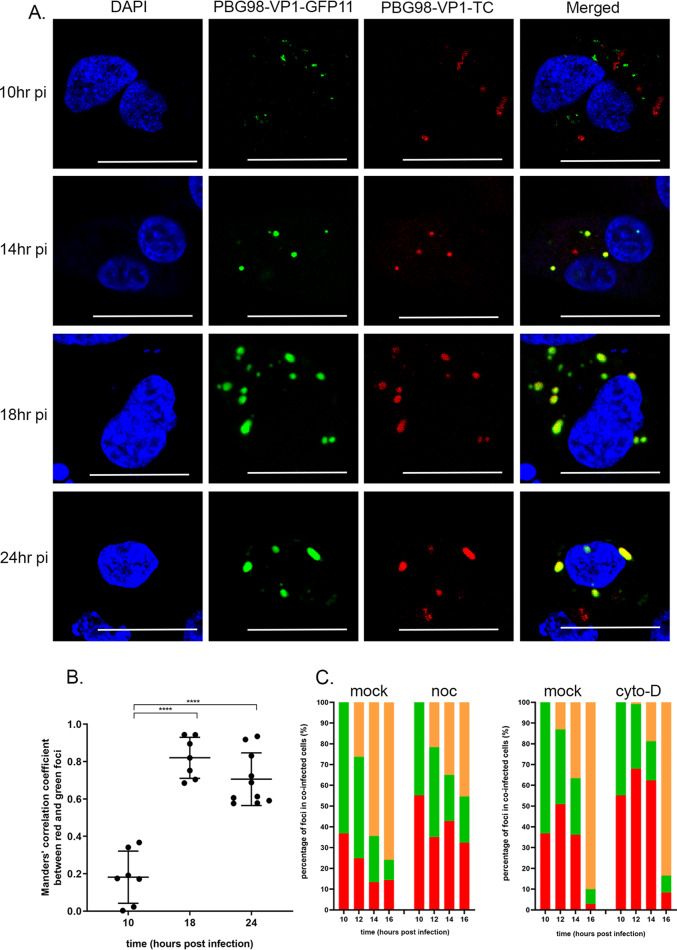
Discrete VFs from different strains of IBDV form in the cytoplasm of coinfected cells and coalesce throughout infection. DF-1 cells were transfected with a plasmid expressing GFP1-10 and coinfected 24 h posttransfection with the PBG98-VP1-GFP11 virus and the PBG98-VP1-TC virus at an MOI of 1. (A) Cells were fixed at 10, 14, 18, and 24 hpi and stained with ReAsH and DAPI. Scale bars, 20 μm. (B) The Mander’s correlation coefficient between red and green foci was plotted for 10 cells at 10, 18, and 24 hpi. The line represents the mean, and error bars represent the SD of the mean. Significance was determined by one-way ANOVA. ****, *P* < 0.0001. (C) The percentages of red, green, and colocalized (yellow) foci were quantified for 30 coinfected cells per time point, and the average was plotted at 10, 12, 14, and 16 hpi in the presence of nocodazole (noc), cytochalasin-D (cyto-D), or DMSO alone (mock).

DF-1 cells were transfected with GFP1-10, coinfected with both PBG98-VP1-GFP11 and PBG98-VP1-TC viruses at an MOI of 1, and then fixed and ReAsH and DAPI stained at 10, 14, 18, and 24 hpi. As in previous experiments, foci at later time points were less numerous and larger than foci at earlier time points, likely as a result of VF coalescence throughout infection (Fig. 8A). At 10 hpi, distinct green and red foci were observed in the cytoplasm of coinfected cells, with little evidence of colocalization, consistent with the presence of discrete VFs from the PBG98-VP1-GFP11 and PBG98-VP1-TC viruses in the cytoplasm. However, at 14, 18, and 24 hpi, there was considerable colocalization between red and green foci (Fig. 8A), consistent with the coalescence of VFs from the PBG98-VP1-GFP11 and PBG98-VP1-TC viruses. Data derived from ImageJ’s colocalization function demonstrated that the levels of colocalization between green and red foci increased from 18.2% at 10 hpi to 70.6% at 24 hpi (*P* < 0.0001) (Fig. 8B). To better define the timing of coalescence between VFs, DF-1 cells were transfected with GFP1-10 and coinfected with both the PBG98-VP1-GFP11 and PBG98-VP1-TC viruses at an MOI of 1 and then fixed and ReAsH and DAPI stained at 10, 12, 14, and 16 hpi. Thirty coinfected cells were imaged per time point, and the average percentage of discrete red and green foci and colocalized (yellow) foci was quantified per cell. In one experiment, an average of 26, 64, and 76% red and green foci colocalized with each other at 12, 14, and 16 hpi, respectively, consistent with the coalescence of VFs from the PBG98-VP1-GFP11 and PBG98-VP1-TC viruses between 10 and 16 hpi. However, nocodazole treatment reduced the percentage of VF coalescence to 22, 35, and 45% at 12, 14, and 16 hpi (Fig. 8C). In another experiment, an average of 13, 37, and 90% foci colocalized with each other at 12, 14, and 16 hpi in the absence of treatment that was reduced to 0.9, 18.8, and 83% at 12, 14, and 16 hpi, demonstrating that cytochalasin D treatment delayed the coalescence of red and green VFs (Fig. 8C). These data were representative of at least three independent experiments and, taken together, demonstrate that during coinfection discrete *Birnaviridae* VFs form in the cytoplasm from each input virus that subsequently coalesce over time in a manner dependent on an intact microtubule network and actin cytoskeleton.

## DISCUSSION

In this study, we produced the first replication-competent tagged-reporter birnaviruses, an IBDV tagged with GFP11 (PBG98-VP1-GFP11) and an IBDV tagged with TC (PBG98-VP1-TC). We used the PBG98-VP1-GFP11 virus to directly visualize IBDV VFs in live infected cells and demonstrated that the VFs were dynamic structures that moved within the cytoplasm and coalesced during the course of infection. These findings are in agreement with others who have found IBDV replication complexes to be located in distinct cytoplasmic puncta (11) that increase in size over the course of infection (13) by imaging fixed cells; however, our data extend these results by imaging live infected cells and characterizing VF movement. The VFs of mammalian orthoreovirus were also recently found to be highly dynamic structures that move and interact in the cytoplasm (15), increasing in size and decreasing in number, consistent with coalescence (33). Therefore, it is possible that the dynamic nature and coalescence of VFs may be a common feature of dsRNA viruses.

We found that destabilization of the microtubule network with nocodazole had a significant impact on IBDV VF size, number, and dynamics throughout infection (Fig. 6 and 7A and Movie S3). Consistent with this observation, Delgui et al. found that when IBDV-infected quail muscle (QM7) cells were treated with nocodazole, more VFs were situated at the periphery of the cytoplasm, failing to traffic to the perinuclear region, unlike in mock-treated cells (11). The distribution of IBDV VFs in our study was less restricted to the perinuclear region in the absence of drug treatment; however, we used DF-1 cells in our experiments, which may account for this discrepancy. Moreover, the distribution of VFs we saw is consistent with data from Dalton and Rodriguez, who also used DF-1 cells (13). Interestingly, *Reoviridae* VFs are also known to hijack the microtubule network during intracellular trafficking (33), and utilization of this cytoskeletal component may be a common feature among dsRNA virus families. We also found that actin filaments colocalized with the periphery of PBG98-VP1-TC VFs and that VF size and speed of movement were significantly reduced following cytochalasin D treatment, suggesting that the actin cytoskeleton is also involved with *Birnaviridae* VF trafficking (Fig. 6 and 7B and Movie S4). Interestingly, VFs have also been observed colocalizing with actin in cells infected with Fowlpox (34) and Bunyamwera (35) viruses, and actin is involved in the internalization, replication, and nonlytic egress of rotavirus (36, 37). IBDV internalization into the cell is also dependent on an intact actin network (32, 38). In order to distinguish between the involvement of actin in IBDV entry and VF movement, cultures were treated with cytochalasin D from 2 hpi, to allow IBDV time to enter the cells prior to treatment. Drug treatment did not significantly alter virus replication (Fig. 6E), demonstrating that the replication of IBDV is not affected by alterations to VF trafficking, consistent with the observations made by Delgui et al. (11).

While the dynamics of VF movement has been previously demonstrated for other dsRNA viruses, our study is the first to image dsRNA VFs in coinfected cells. We demonstrated that during coinfection, discrete VFs from each input virus initially formed in the cytoplasm that subsequently coalesced over time, with the proportion of VFs containing both GFP11- and TC-tagged VP1 proteins increasing throughout the replication cycle (Fig. 8). The diversity of RNA viruses is increased through recombination and/or reassortment in cells coinfected with multiple strains. In order for this to occur, the genome from two or more infecting viruses must reach the same intracellular compartment. Using fluorescently tagged vaccinia viruses, Paszkowski et al. demonstrated that during coinfection, distinct VFs from two input viruses formed in the cytoplasm and subsequently merged. These authors concluded that the VFs acted as a barrier to genomic mixing and that the merger of VFs was necessary for recombination (39). It has also been proposed that the coalescence of VFs must take place in order for reassortment of some dsRNA viruses to occur (40), in contrast to viruses such as influenza, where genome segments coalesce en route to the plasma membrane (41) and where reassortment is not restricted by VF compartmentalization. Our data are consistent with this being the case for the *Birnaviridae* as the genome was compartmentalized to VFs (Fig. 2), and the fluorescent signal from FISH probes designed to bind viral mRNA was also colocalized with the VFs, although we cannot rule out the probes also binding to the positive strand of the dsRNA genome, as well as the mRNA.

Our study is not without limitations; for example, the fluorescent signal was only detected from 8 hpi and was only bright enough to be reliably imaged from 10 hpi for either the GFP11- or the TC-tagged viruses. At 10 hpi, numerous small VFs were observed (Fig. 3A), even in cells infected at a very low MOI, implying that the VFs are not the product of one cell being infected with multiple infectious viruses. The molecular basis of this observation remains unknown, and, as we were unable to study events earlier than 8 hpi, this remains beyond the scope of the present study.

We were also unable to rescue a replication competent PBG98-VP1-GFP virus. However, GFP11 and GFP1-10 come together to give a fluorescent signal, indicating that the VP1-GFP molecule is functional. The crystal structure of the birnavirus VP1 has been determined (42, 43) and our data demonstrate that the C-terminal extension is able to tolerate the insertion of a tag, without blocking the function of the active site. However, the tagged viruses were both attenuated compared to the recombinant wt PBG98 strain (Fig. 1E and F). This could be because the presence of the tag negatively affected the function of the VP1 protein or because the presence of the nucleotide sequence that encoded the tag made the genome segment less efficient at being packaged. Moreover, the GFP11-tagged virus was more attenuated and less stable than the TC-tagged virus, which correlates with the length of the tag. Investigating the reason for the differences in attenuation was beyond the scope of this project. In addition, some VP1 is packaged into the virion, while the rest remains in the cytoplasm, and some VP1 is bound to the dsRNA genome, while the rest remains unbound. It is unknown whether the VP1-GFP is packaged into the virion, or whether it is bound to the genome. Discerning these differences was beyond the scope of this project. Since the TC tag was less attenuated, it can be considered an accurate representation of IBDV-derived VFs in infected cells. However, the fluorescence produced by the staining of the TC tag with ReAsH was more prone to bleaching than the GFP signal, so the GFP11-tagged IBDV strain was a more attractive candidate for live cell imaging over long time courses. In order to detect a green signal, it was necessary for cells to be both successfully transfected with GFP1-10 and infected with the GFP11-tagged virus. While this approach is adequate for imaging experiments, we are currently establishing a DF-1 cell line that stably expresses the GFP1-10 molecule for additional studies.

In order to study birnavirus reassortment, we attempted to rescue an IBDV with a tag on the C terminus of Segment A; however, this virus did not rescue. Segment A is translated as a polyprotein encoding VP2-VP4-VP3, which is subsequently cleaved (44). The C terminus of VP3 has recently been shown to be essential for function (45), which might explain why this was an unsuitable position for the tag; however, given that there are cleavage sites at the VP2-VP4 and VP4-VP3 junctions, it may be difficult to generate a tagged segment A. Finally, since IBDV can incorporate multiple copies of VP1 into one virion (46), we reasoned that during coinfection some virions would contain VP1-TC, as well as VP1-GFP11, and that reassortment could be quantified by determining the number of red, green, and yellow plaques from the supernatant of coinfected cultures. However, unfortunately, the tagged viruses failed to form readily discernible plaques, and it was not possible to complete these experiments. Moreover, we were cautious about quantifying reassortment using the tagged viruses owing to differences in biological fitness between the GFP11- and TC-tagged viruses.

Taken together, our data provide the first experimental demonstration of VF coalescence for the *Birnaviridae* and, to our knowledge, the first evidence of coalescence between VFs in a coinfected cell for any dsRNA virus family. We speculate that VF coalescence is required for *Birnaviridae* reassortment and suggest that the potential for IBDV reassortment occurs late in the viral replication cycle, since VF coalescence between GFP11- and TC-tagged viruses did not occur until after 10 hpi. This study provides new information that adds to our understanding VF trafficking that could have implications for the molecular basis of dsRNA virus reassortment.

## MATERIALS AND METHODS

### Cell-lines and antibodies.

DF-1 cells (chicken embryonic fibroblast cells, ATCC number CRL-12203) were sustained in Dulbecco modified Eagle medium (DMEM; Sigma-Aldrich, Merck, UK) supplemented with 10% heat-inactivated fetal bovine serum (hiFBS; Gibco, Thermo Fisher Scientific, UK). The primary antibodies used in this study were raised against tubulin (Santa Cruz Biotechnology, UK), dsRNA (English & Scientific Consulting Kft.), and VP3 (47). In all immunofluorescent assays, primary antibodies were diluted 1:100 and secondary antibodies conjugated to Alexa 488 or Alexa 568 (Invitrogen, Thermo Fisher Scientific) were diluted 1:500 in a solution of bovine serum albumin (BSA; Sigma-Aldrich).

### Plasmids and recombinant viruses.

The sequences of segments A and B of the cell culture-adapted IBDV strain PBG98, including the 5′ and 3′ noncoding regions (GenBank accession numbers MT010364 and MT010365), were flanked by self-cleaving ribozymes (a hammerhead ribozyme upstream and a hepatitis delta ribozyme downstream [48]). The whole sequence was ordered (GeneArt, Thermo Fisher Scientific) and cloned into a pSF-CAG-KAN vector (Sigma-Aldrich) using restriction enzyme pairs KpnI/NheI (segment A) and SacI/XhoI (segment B; New England Biosciences, UK) to make two “reverse genetics” plasmids (pRGs), pRG-PBG98-A and pRG-PBG98-B. DF-1 cells at 70% confluence were transfected with both plasmids with Lipofectamine 2000 (Invitrogen, Thermo Fisher Scientific) in order to rescue the recombinant PBG98 virus. Three alanine residues were added to the N terminus of the GFP11 tag as a linker and a stop codon was added to the C terminus to make the amino acid sequence AAARDHMVLHEYVNAAGIT-Stop. The nucleotide sequence encoding this (GCCGCCGCCCGCGATCACATGGTCCTGCACGAGTACGTGAACGCCGCCGGGATCACTTAG) was added to the 3′ end of the coding region of segment B, which encodes VP1, prior to the 3′ noncoding region to make the plasmid pRG-PBG98-B-GFP11 (GeneArt, Thermo Fisher Scientific). DF-1 cells were cotransfected with this plasmid and pRG-PBG98-A to generate the recombinant virus PBG98-VP1-GFP11. The tetracysteine tag (amino acid sequence CCPGCC, nucleotide sequence TGTTGTCCTGGCTGTTGCTGA) was incorporated into the same region as the GFP11 tag to make the pRG-PBG98-B-TC plasmid (GeneArt, Thermo Fisher Scientific), and DF-1 cells were cotransfected with this plasmid and pRG-PBG98-A to make the recombinant virus PBG98-VP1-TC.

### Visualizing virus infection.

To visualize PBG98-VP1-GFP11 virus infection, DF-1 cells were seeded onto coverslips (TAAB, UK) in 24-well plates (Falcon, Corning, UK) at a density of 1.6 × 10^5^ per well and transfected with GFP1-10 using Lipofectamine 2000 24 h prior to infection with the PBG98-VP1-GFP11 virus. Unless otherwise stated, the cells were infected at an MOI of 1. To visualize PBG98-VP1-TC virus infection, live infected cells were stained with the TC-ReAsH II In-Cell tetracysteine tag detection kit (Invitrogen, Thermo Fisher Scientific) according to the manufacturer’s instructions.

### Immunofluorescence microscopy.

Cells were fixed with a 4% paraformaldehyde solution (Sigma-Aldrich) for 20 min, permeabilized with a solution of 0.1% Triton X-100 (Sigma-Aldrich) for 15 min, and blocked with a 4% BSA solution for 30 min. The cells were then incubated with the appropriate primary antibody for 1 h at room temperature. Cells were washed with phosphate-buffered saline (PBS) and incubated with the corresponding secondary antibody for 1 h at room temperature in the dark. The cells were again washed and incubated in a solution of DAPI (4′,6′-diamidino-2-phenylindole; Invitrogen, Thermo Fisher Scientific). The cells were washed in water, mounted with Vectashield (Vector Laboratories, Inc., CA), and imaged with a Leica TCS SP5 confocal microscope. To image actin, phalloidin-Alexa Fluor 488 (Thermo Fisher Scientific) was added directly to cells in PBS after blocking, followed by incubation at room temperature for 25 min prior to DAPI staining.

### Live cell imaging.

DF-1 cells were seeded into a chambered 1.0 borosilicate cover glass slide (Nunc, Lab-Tek, Sigma-Aldrich, Merck) at a density of 8 × 10^4^ per well and cultured in 1 ml of DMEM supplemented with 10% hiFBS. Cells were transfected with GFP1-10 and then infected with the PBG98-VP1-GFP11 virus at 24 h posttransfection. Cells were maintained in Leibovitz L-15 media without phenol red (Gibco, Thermo Fisher Scientific) during live cell imaging experiments. Unless otherwise stated, 10-Z stacks were imaged every 4 min for a minimum of 2 h using a Leica TCS SP8 confocal microscope.

### Fluorescence *in situ* hybridization.

A total of 48 Stellaris FISH probes (Biosearch Technologies, CA) were designed to bind mRNA from segment A, and 48 were designed to bind mRNA from segment B of PBG98 using the RNA FISH probe designer (Biosearch Technologies). Probes designed against segment A were conjugated to Quasar 570 Dye and probes designed against segment B were conjugated to Quasar 670 Dye. DF-1 cells were transfected with GFP1-10 and infected with the PBG98-VP1-GFP11 virus, and probes were hybridized according to the manufacturers’ instructions at 24 hpi. Briefly, the cells were fixed in a formaldehyde solution and permeabilized in ethanol, and probes were diluted in hybridization buffer containing deionized formamide. Probes were hybridized to the samples in a humidified chamber at 37°C for at least 4 h, and samples were washed, stained with DAPI, and imaged. Although the probes were designed to bind viral mRNA, cross-reactivity to the positive strand of the dsRNA viral genome could not be ruled out.

### Virus titration.

Samples were titrated in 96-well plates (Falcon, Corning, UK) seeded with DF-1 cells at a density of 4 × 10^4^ cells per well. Wells were then infected in quadruplicate with a 10-fold dilution series of viral supernatant. After 5 days, the wells were inspected for signs of cytopathic effect, and the virus titer was determined from the TCID_50_ according to the method of Reed and Muench (49).

### Virus growth curves.

DF-1 cells were seeded into 24-well plates at a density of 1.6 × 10^5^ cells per well in triplicate for each time point. The next day, cells were infected with either PBG98-VP1-GFP11, PBG98-VP1-TC, or PBG98 viruses at an MOI of 0.01. Cell supernatant was then collected at 24, 48, and 72 hpi and titrated as described by Reed and Muench (49).

### Passage stability.

DF-1 cells were seeded into 24-well plates at a density of 1.6 × 10^5^ per well and maintained in 900 μl of media overnight. The cells were subsequently infected with 100 μl of the supernatant collected from the previous passage. After 24 h, the supernatant was collected and frozen at –80°C until the next passage. Both fluorescently tagged IBDV-viruses were passaged ten times, whereupon DF-1 cells were infected with the supernatant from every passage in order to image infected cells 20 hpi. The cells were transfected with GFP1-10 prior to infection with PBG98-VP1-GFP11 supernatants and were stained with ReAsH subsequent to infection with PBG98-VP1-TC supernatants.

### Drug treatment.

DF-1 cells were seeded onto coverslips and infected with the PBG98-VP1-TC virus. Cultures were subsequently treated with either cytochalasin D or nocodazole (Sigma-Aldrich, Merck). Drugs were dissolved in dimethyl sulfate (DMSO; Sigma-Aldrich, Merck) and diluted with media to final concentrations of 1 and 10 μM, respectively. Infected cultures were treated with drugs from 2 hpi until the end of the experiment. An equivalent volume of DMSO was added to the culture media of mock-treated control cells.

### Image quantification and statistics.

Virus titers were compared by one-way analysis of variance (ANOVA) and a Tukey’s multiple-comparison test following a Shapiro-Wilk normality test to confirm whether the data followed a normal distribution for parametric or nonparametric testing, using Prism 7 (GraphPad). The mean area and the number of VFs were calculated using the surface tool in Imaris 9 software (Bitplane, Oxford Instruments, UK), and the number and size of foci were compared using a Kruskal-Wallis one-way ANOVA using Prism. An overlap coefficient (an alternative to the Pearson’s correlation coefficient created by Manders et al.) was used to describe colocalization using ImageJ software (National Institutes of Health). Images were only considered in the analysis if they had a Coste’s significance level of 0.95 or above (17), and significance was determined by a one-way ANOVA using Prism. For live cell imaging, statistics such as displacement, velocity, and the number of fusion and fission events were calculated using the ImageJ plugin TrackMate, and statistical significance was determined by a one-way ANOVA using Prism (*, *P* < 0.05; ***, *P* < 0.001; ****, *P* < 0.0001).

### Data availability.

The complete sequences of segments A and B of infectious bursal disease virus strain PBG98 are available in GenBank under accession numbers MT010364 and MT010365.

## Supplementary Material

Supplemental file 1

Supplemental file 2

Supplemental file 3

Supplemental file 4

Supplemental file 5
