# Supplementary material for: Discrete Virus Factories Form in the Cytoplasm of Cells Coinfected with Two Replication-Competent Tagged Reporter Birnaviruses That Subsequently Coalesce over Time
Source: J Virol. 2020 Jun 16;94(13):e02107-19. doi: 10.1128/JVI.02107-19 (PMC7307154; doi:10.1128/JVI.02107-19)
Supplement: Supplemental file 1 [file JVI.02107-19-s0001.pdf]

Movie legends

Movie S1. VFs coalesce in the cytoplasm of infected cells between 16 and 18 hpi. DF-1 cells were transfected with a plasmid expressing GFP1-10 and infected 24 hours post-transfection with the PBG98-VP1-GFP11 virus at an MOI of 1. One live infected cell was imaged from 16-18 hpi; one image obtained every 4 minutes.

Movie S2. VFs coalesce in the cytoplasm of infected cells between 22 and 25 hpi. DF-1 cells were transfected with a plasmid expressing GFP1-10 and infected 24 hours post-transfection with the PBG98-VP1-GFP11 virus at an MOI of 1. One live infected cell was imaged from 22-25 hpi; one image obtained every 4 minutes.

Movie S3. VF coalescence is dependent on an intact microtubule network. DF-1 cells were transfected with a plasmid expressing GFP1-10 and infected 24 hours post-transfection with the PBG98-VP1-GFP11 virus at an MOI of 1. Cells were treated with nocodazole from 2 hpi. One live infected cell was imaged from 20-22 hpi; one image obtained every 4 minutes.

Movie S4. VF coalescence is dependent on an intact actin cytoskeleton. DF-1 cells were transfected with a plasmid expressing GFP1-10 and infected 24 hours post-transfection with the PBG98-VP1-GFP11 virus at an MOI of 1. Cells were treated with cytochalasin-D from 2 hpi. One live infected cell was imaged from 20-22 hpi; one image obtained every 4 minutes.
